# #FakeMeat: How big a deal will animal meat analogs ultimately be?

**DOI:** 10.1093/af/vfy011

**Published:** 2018-07-19

**Authors:** Lisa M Keefe

**Affiliations:** Meatingplace, MTG Media Group, Chicago, IL

**Keywords:** Impossible Burger, Beyond Meat, Memphis Meats, ground beef, plant-based

**Implications**

- New technologies behind plant-based meat analogs and lab-cultured muscle tissue have captured the attention of the public and investors. While plant-based meat substitutes exist in a market that has been around for decades, the new technologies are significantly different—and, most agree, significantly better tasting than earlier products made primarily of soy protein concentrate and wheat gluten—and so represent a sizable potential challenge to traditional, harvested meat producers and processors.
- The meat industry is operating from a position of significant strength, however, being well established, extremely cost-effective, and many times larger than the nascent meat analog and cultured tissue markets.
- The meat industry should prepare to move and change along with demographic shifts toward smaller households (smaller portions and packaging), consumers who have less time for food preparation (premarinated, precooked meat and meat ingredients, further processing into easily used forms, such as dices and cubes, for stews and other dishes that use meat as an ingredient rather than as center of the plate), emphasizing niche markets with desirable attributes, such as Organic and no-antibiotics-ever production.
- The meat industry already has engaged the USDA in an effort to clarify, as quickly as possible, how the new technologies will be treated in a regulatory manner, which will significantly influence how the products can be labeled and marketed, and even produced.
- The next 3 to 5 yr will be key to how traditional animal-harvested meat products and meat analogs and lab-cultured tissue will coexist in the consumer market, and whether the traditional meat industry will retain, or cede, market share to the new technologies.

Impossible Burger, Beyond Meat, and Memphis Meats—companies that produce, or promise to produce, a meat-like product to rival actual ground beef in taste, mouthfeel, and overall enjoyability dominated food news in 2017 and into 2018. And why not? Despite its generally made-from-scraps, low-on-the-totem-pole position among beef products, ground beef in burgers and other forms constitutes some 60% of overall beef consumption in the United States, a market worth about $44.5 billion a year (Rutherford, 2014; USDA ERS, 2017).

As well, the companies and the individuals investing in this developing area of food production are themselves headline-makers, including Bill Gates, Leonardo DiCaprio, Sergey Brin, Tyson Foods, and Cargill.

Animal welfare activists and vegetarian/vegan proponents have hailed the introduction of these products, saying, “thanks to science, we can have our (vegan) cake and save the planet, too.” (People for the Ethical Treatment of Animals, 2017). They are building a niche in parts of the market traditionally occupied by meat products, primarily in burger sandwiches. Beyond Meat also has introduced a meat-free chicken strip product through retail stores.

The analog companies and activists predict that these new products are the beginning of the end of meat consumption as historically harvested from livestock. However, countervailing social, economic, and environmental factors are poised to change that storyline once the initial excitement over the food industry’s shiny, new idea has died down.

## Meat Substitutes’ Long History

The new meat substitute products are far from the first to hit store shelves. The first no-meat burger product available commercially debuted in the United Kingdom in 1982 (Smith, 2014). In the United States, Gardenburgers were first sold at retail stores in 1992, and Boca Burgers beginning the year after that (Smith, 2014). The 21st-century versions are quite different from their predecessors, which are made mostly of soy protein concentrate and wheat gluten. Whereas Gardenburger, Boca Burgers, and Morningstar Farms products are marketed to vegetarians with the vegetable base being the primary attribute, Impossible Burger and Beyond Meat’s products are marketed to meat-eaters on the basis of their resemblance to meat.

Impossible Burger, for example, is made of wheat and potato protein, coconut oil, water, added vitamins and minerals, and leghemoglobin from soy, which gives the burger its meat-like mouthfeel and taste. The product’s manufacturer, Impossible Foods, has focused on distributing it through restaurants, from Momofuku Nishi in New York City to White Castle.

Beyond Burger, meanwhile, is made of a pea protein isolate and various oils, including coconut oil, with cellulose and potato starch and other ingredients to create the right texture and flavor. Part of Beyond Burger’s marketing pitch is that the patties “bleed” like a beef burger, thanks to the use of beet juice extract.

**Figure:**
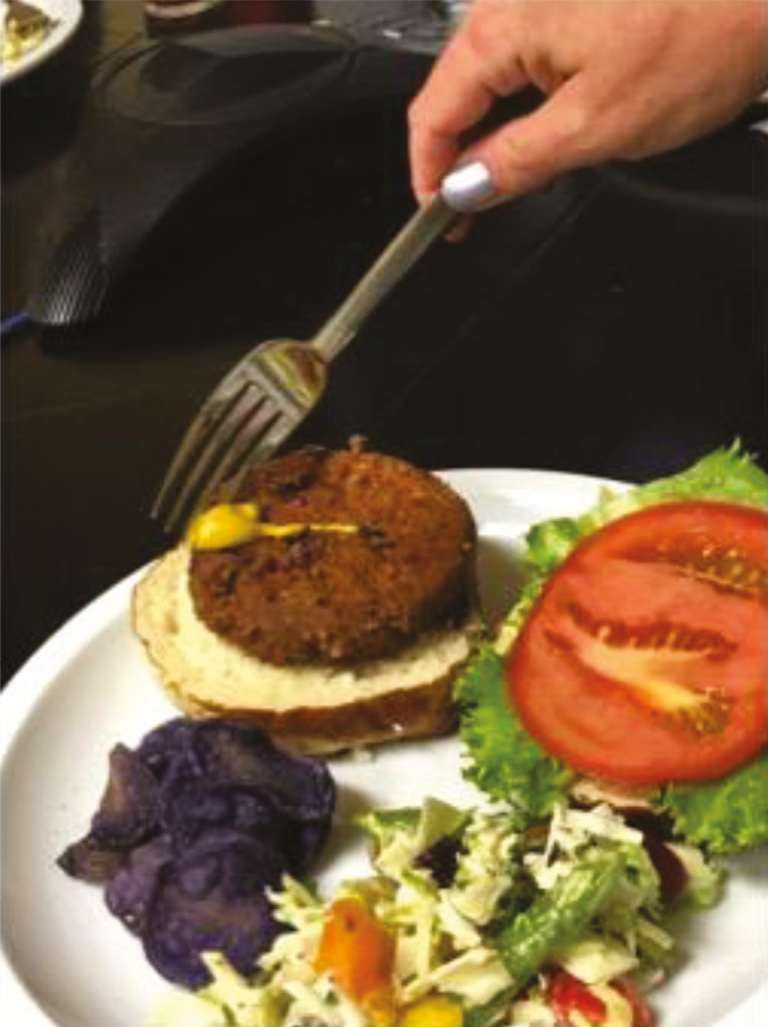
Meatingplace editorial staff tried Beyond Burger for lunch one day. (source: Lisa M. Keefe)

Similarly, in spring 2018, a Hong Kong–based company called Right Treat announced it would offer Omnipork, a vegan, plant-based version of pork, created especially for the Asian market. Omnipork is a “proprietary blend of plant-based protein from pea, non-GMO soy, shiitake mushroom and rice,” according to the company’s website. The product still needs regulatory approval in China to begin commercial distribution.

Beyond Burger’s manufacturer, Beyond Meat, also makes sausage and chicken analog products, sold through retail stores and—a key factor—merchandised in the meat case itself or nearby.

The total meat substitutes market is expected to be around $6.43 billion in 2023, according to a Markets and Markets press release (2018), although that figure includes soy, tofu and seitan products, as well as the newer iterations. That is a mere speck compared with meat consumption in the United States, however, proponents point out that the meat substitutes market is growing at a compound annual growth rate of about 6.3% annually, whereas meat consumption in the United States overall is less than half that rate. (Widmar, 2018; Table 1; United Nations, FAO, 2003).

**Table 1.** Projected increase in meat consumption, by region (UN FAO, 2003)

Food consumption of meat							
	1964/66	1974/76	1984/86	1994/96	1997/99	2015	2030
	kg per capita, carcass weight equivalent						
World	24.2	27.4	30.7	34.6	36.4	41.3	45.3
Developing countries	10.2	11.4	15.5	22.7	25.5	31.6	36.7
excl. China	11.0	12.1	14.5	17.5	18.2	22.7	28.0
excl. China and Brazil	10.1	11.0	13.1	14.9	15.5	19.8	25.1
Sub-Saharan Africa	9.9	9.6	10.2	9.3	9.4	10.9	13.4
Near East/North Africa	11.9	13.8	20.4	19.7	21.2	28.6	35.0
Latin America and the Caribbean	31.7	35.6	39.7	50.1	53.8	65.3	76.6
excl. Brazil	34.1	37.5	39.6	42.4	45.4	56.4	67.7
South Asia	3.9	3.9	4.4	5.4	5.3	7.6	11.7
East Asia	8.7	10.0	16.9	31.7	37.7	50.0	58.5
excl. China	9.4	10.9	14.7	21.9	22.7	31.0	40.9
Industrial countries	61.5	73.5	80.7	86.2	88.2	95.7	100.1
Transition countries	42.5	60.0	65.8	50.5	46.2	53.8	60.7
Memo item							
World excl. China	28.5	32.6	34.3	34.1	34.2	36.9	40.3
World excl. China and transition countries	26.5	29.0	30.6	32.4	33.0	35.6	39.1

Source: UN Food and Agriculture Organization.

## Silicon Valley Meets Meat

“Cellular agriculture” is a major area of new product development in meat alternatives, and the basis for a number of corporate start-ups (Dance, 2017; Nanalyze, 2017) that has arisen from advances in the field of tissue engineering. For example, these methods are applied to produce sheets of cultured epithelial tissue that are applied as engineered skin to help heal burn victims’ wounds. Engineered skeletal muscle can be useful for regenerative medicine, for treating muscle injuries or muscular defects, or for engineering meat (Langelaan, et al., 2010). Variously referred to as “cultured meat”, “synthetic meat”, “clean meat”, “lab-grown meat”, or “in vitro meat”, the process begins with muscle stems cells (myoblasts) obtained from skeletal muscle biopsies of cattle—or, hogs or poultry. The cells are grown in flasks, bathed in a nutrient-dense culture medium where they multiply, forming strands of muscle tissue (reviewed in Arshad et al., 2017; Post and Hocquette, 2017; Mattick, 2018).

Unlike the meat analogs, the lab-grown or cultured muscle tissue has not reached the point of commercialization, largely due to technical limitations. Taste-test events have been held, most notably in 2013 at the University of Maastricht, where Prof. Mark Post served a cultured beef burger with a sunken cost, in total, of about $325,000 (Jamieson and Boyle, 2013). Testers reported it had a “rather intense flavor” although it was dry, not juicy. In an interview, Post indicated the culture process took 3 mo to generate 20,000 muscle fibers to produce a 5 ounce “hamburger,” and still requires the use of fetal bovine serum (Papadopoulos, 2017).

More recently, Memphis Meats—which, despite its name, is based in San Francisco—has taste-tested “tenders” made from lab-grown chicken tissue, at an approximate cost of $6,000 per pound.

**Figure:**
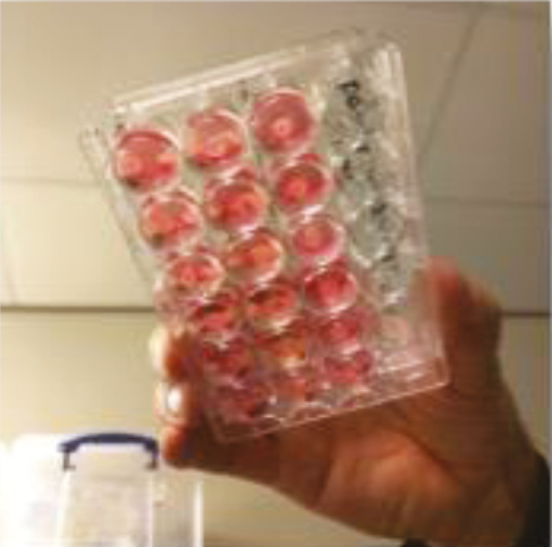
Lab-grown, or cultured, muscle tissue is grown in nutrient-dense media. (source: New-Harvest.org)

Cultured muscle tissue, with its patina of technology and biomedicine, has attracted Silicon Valley–worthy investors, including Bill Gates, Suzy Welch, Richard Branson, Kimbal Musk, and Sergey Brin. The number of companies around the globe pursuing this market is proliferating: In addition to Memphis Meats and MosaMeat (a company that grew out of Maastricht University’s research); SuperMeat, FutureMeat Technologies, and Aleph Farms, allbased in Israel; JUST Inc. and Mission Barns Inc. in the U.S., have emerged. Considering the level of investment and rapid product development, some speculate that these laboratory goods could be on the market in 24 to 36 mo (Dance, 2017). However, this optimistic projection of time to market will require that several technical obstacles related to the culture process and are overcome (Mandelbaum, 2017).

## What Is in a Name?

The arrival of plant-based meat analogs and the prospect of the commercialization of the lab-grown technologies are roiling the meat industry. The first front for attack is the nomenclature. For example, vegan activists have coined the phrase “clean meat” for lab-grown muscle tissue because the final product does not depend on the slaughter of an animal, does not introduce all the potential contaminants present on a living animal, such as feces and pathogens. The production process is as close to being sanitary as food production could be, with potential contaminants possible only from the raw ingredients, original muscle samples, culture medium ingredients, or manufacturing process.

In early 2018, the phrase “clean meat” was migrating rapidly from its most common usage in activist and environmental media to mainstream outlets, including CNBC, *The Washington Post*, and *Wired* magazine. Its easy adoption in everyday use—and the tacit implication that anything else is “not clean” meat—sufficiently spooked the traditional meat-processing industry, that in early 2018 different parties launched campaigns at the federal and state level to prevent lab-grown tissue from being considered “meat” in regulatory code, or called “meat” in advertising and labels. Alternatively, if it is to be designated “meat,” parties have asked that it be subject to the same level of regulatory scrutiny as the traditional animal-harvesting meat industry.

In February 2018, the United States Cattlemen’s Association (**USCA**) submitted a petition to the USDA’s Food Safety and Inspection Service (**USDA FSIS**) asking the agency for rulemaking on beef labeling to clarify for consumers what is beef derived from cattle and “beef” products created in a laboratory (Johnston, 2018).

 “Consumers depend upon the USDA FSIS to ensure that the products they purchase at the grocery store match their label descriptions,” USCA President Kenny Graner said in a news release (Johnston, 2018) at the time. “We look forward to working with the agency to rectify the misleading labeling of ‘beef’ products that are made with plant or insect protein or grown in a petri dish.”

The National Cattlemen’s Beef Association and the National Farmers Union followed suit in April 2018. Also, in April 2018, legislators in the state of Missouri House of Representatives introduced a bill prohibiting “misrepresenting a product as meat that is not derived from harvested production livestock or poultry.” As of mid-May, 2018, the state House had passed the bill and it was slated to be considered by the Missouri Senate (Missouri HB 2607).

## Not so Fast

**Figure 1.**
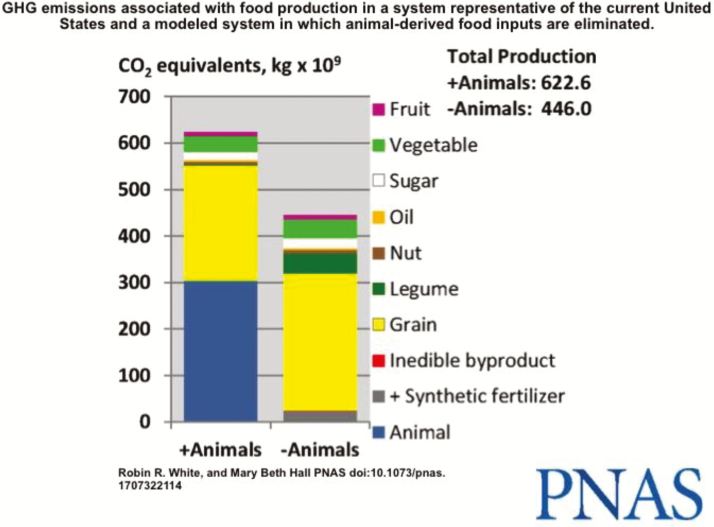
Eliminating all food animals from the environment would not have as great an effect as many proponents would like the public to think, according to research at Virginia Tech and USDA (White and Hall, 2017).

As enthusiasm for the new animal-harvested meat substitutes has grown, so has research into the ripple effects of such a fundamental change in the food chain. While the products’ proponents paint a picture of a future with less greenhouse gases and fewer, presumably happier livestock animals, a study done by USDA’s Agricultural Research Service and Virginia Tech University calculated some of the less beneficial results (Figure 1; White and Hall, 2017).

Setting out to explore the nutritional and greenhouse gas impacts of removing animals from U.S. agriculture, researchers found that a complete shift away from food animal production would present major challenges to meeting America’s nutritional needs. With no meat, milk, eggs, fish, or cheese in the American diet, the U.S. population would not receive enough of several different essential dietary nutrients from the foods they eat, according to the study results.

“Different types of carefully balanced diets — vegan, vegetarian, omnivore — can meet a person’s needs and keep them healthy, but this study examined balancing the needs of the entire nation with the foods we could produce from plants alone. There’s a difference between what’s possible when feeding one person versus feeding everyone in the U.S.,” said ARS scientist Mary Beth Hall in an interview (Kelly, 2017).

Eliminating food animals would increase human deficiencies in calcium, vitamins A and B12, and some important fatty acids. Fatty acids help to reduce cardiovascular disease and improve cognitive function and vision in infants. Animal food products are the only available, nonsupplemental sources of some fatty acids and vitamin B12 (Figure 1; White and Hall, 2017).

A plant-only diet also would require individuals to eat more food and more daily calories to meet their nutritional needs because the available foods from plants are not as nutrient dense as foods from animals, the researchers said.

The scientists found that shifting land usage from food animal production to food crop production would increase the total U.S. food supply by 23%. Because much of that land is unsuitable for high-value crops, most of the additional food produced would include high-calorie crops like corn and soybeans. They also determined that eliminating food animals from U.S. production would reduce greenhouse gas emissions, but not by the full 49% of agricultural emissions that animals currently contribute. Rather, greenhouse gas emissions from agriculture would drop by 28% without farmed animals because of increases associated with producing additional food crops and the use of more synthetic fertilizer to replace manure. That would represent a drop of only about 2.6% of total U.S. emissions (White and Hall, 2017).

## Increasing Consumption

**Figure 2.**
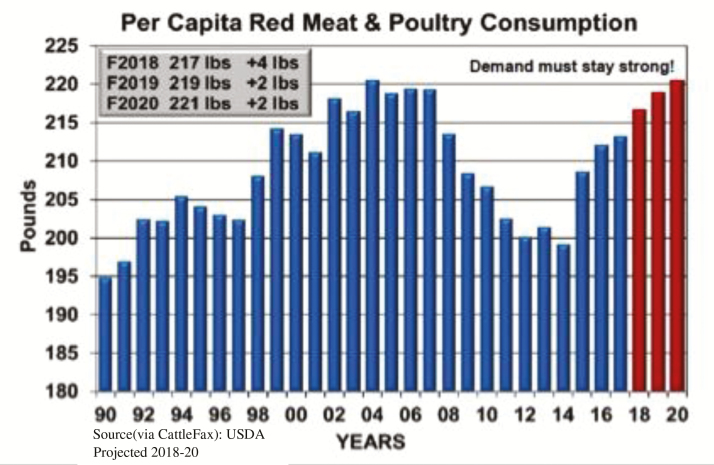
Meat consumption in the United States is expected to hit record levels in 2018 (USDA ERS, 2017).

Meanwhile, although the hype is about meat substitutes, consumption of traditionally harvested meat products is growing, including in the United States (Sawyer, 2016). In fact, USDA’s Economic Research Service projected a record amount of beef, pork, and poultry consumption by U.S. consumers in 2018 (Figure 2; USDA ERS, 2017).

USDA projected beef per capita disappearance (the volume of meat and poultry production that remains for domestic use after subtracting net trade and changes in cold storage volumes) at 59.4 pounds in 2018, up from 56.8 pounds in 2017 and 55.6 pounds in 2016 (USDA ERS, 2018). Per capita pork disappearance was projected at 52 pounds in 2018, up from 50.2 pounds in 2017 and 50.1 pounds in 2016. U.S. broiler disappearance was pegged at 92.4 pounds per capita in 2018, up from 90.7 pounds in 2017 and 89.8 pounds in 2016. Turkey per capita disappearance was forecast at 16.5 pounds, up from 16.4 pounds in 2017, but down from 16.6 pounds in 2016.

The most important factors driving per capita disappearance for the year, according to USDA were projected increases in year-over-year production of beef (up 6.1%), pork (up 5.4%), and broiler meat (up 2.1%). This, despite a slow decline in domestic red meat consumption beginning in the 1970s, and a decline in meat consumption overall, including poultry, in the 2000s and particularly during and just after the Great Recession (USDA ERS, 2017).

Globally, meat consumption has risen since the 1970s, largely mirroring the economic growth of developing countries. Including data from China and Brazil, which have an outsized effect on the overall trend, the per capita meat consumption in the developing countries increased from 11.4 kg per capita annually in the early 1970s to 25.5 kg in 2003 and 36.7 kg projected in 2030.

Global growth has fueled a robust export business in the U.S. meat industry: In 2017, 12.9% of U.S. beef production, including offal, was exported, along with 26.6% of U.S.-produced pork, according to the U.S. Meat Export Federation (USDA ERS, 2017). About 17% of U.S. broiler production was exported in 2017, according to the National Chicken Council (2018).

In developing countries, demographic shifts are bringing their population profiles more in line with that of developed nations. Specifically, a larger percentage of residents in developing countries now live in urban areas than live in rural areas, two trend lines that just recently crossed one another.

A more urban population needs to have its food supplied by the dwindling number of people who are farming. Technology has benefitted the trend by, on the one hand, increasing the volume of shelf-stable meat products, such as meat snacks, which then can be transported to areas with little infrastructure. Alternatively, much as cell phones were adopted much more quickly in developing countries than in developed ones—precisely because of the lack of land-line telecommunications infrastructure—so are power sources such as solar panels becoming quickly adopted in areas with no power infrastructure.

For example, a small, even single, solar cell on the roof of a hut can power a refrigerator inside, making it possible to keep fresh food cold regardless of location or climate.

Export trends would be significantly disrupted if the United States and other countries pursue the kind of protectionist agenda that has been in the headlines at the beginning of 2018. For one, tensions with China has led that country to slap a 25% retaliatory tariff on pork from the United States. China is the No. 2 export market for U.S. pork, after Mexico, and bought more than 1 billion pounds of the meat in 2017 (National Pork Board, 2018).

Along with plans to renegotiate key assumptions of the North American Free Trade Agreement, which has boosted the U.S. meat industry since its passing in 1993, trade issues have become a source of volatility in the meat markets. For example, prices would have to drop significantly if the high levels of production across species in the United States ended up having to be absorbed by the domestic market.

## Meat as an Ingredient

**Figure 3.**
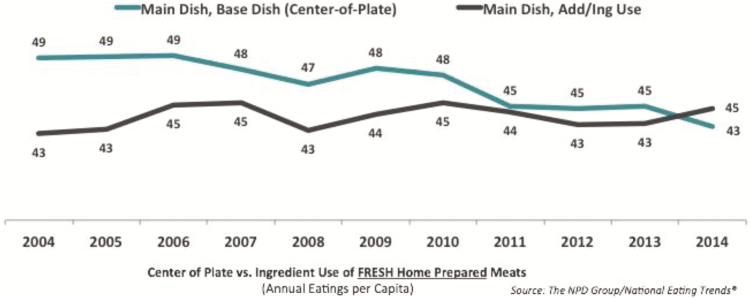
More consumers are using meat as an addition to or ingredient in dishes, rather than as a center-of-the-plate item (Produced from analysis of The NPD Group database, 2014).

Quite aside from the growth in global demand and the uncertainty around trade relationships, how meat is being consumed, especially in the United States, is different now than in decades past. Diners are more likely to eat meat as an addition or ingredient to a dish than as a center-of-the-plate feature in a meat-and-two-veg meal. The trend includes ground beef in burgers, meatloaf, tacos and the like, as well as diced and cubed meats in chili, salad, and stews. A keen interest in ethnic cuisines among younger consumers—millennials and so-called Gen Z—also feeds the demand for meat as an ingredient, as few cuisines outside the Anglo cultures plate the meat separately from the other ingredients (Figure 3).

**Figure:**
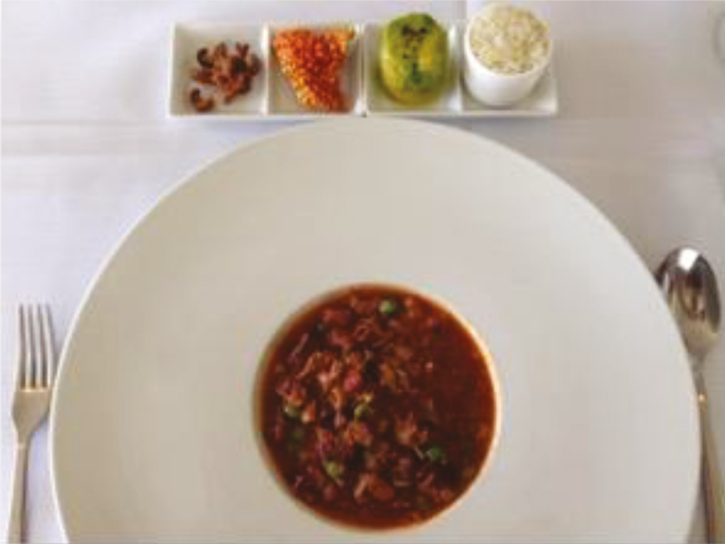
Meatingplace editorial staff tried Beyond Meat is used more often as an ingredient in dishes such as stews. (source: Getty Images stock photo)

As well, a huge demographic shift in the United States and in other developed countries is toward smaller households: In 1940, only 7.8% of U.S. households were single-person homes, but by 2014 that percentage had climbed to 28% (Keefe, 2016). Single households include not only never-marrieds but empty-nester boomers, divorcees, surviving spouses, especially no-longer or never-married career women of all ages.

On the production side, cattlemen are beginning to look at the profitability of raising more cattle aimed at the lower end of Choice and Select grades, with an eye on using that meat to feed the demand for ground beef. A so-called “grinding animal” could be left on forage for a longer time combined with a shorter grain-feeding period, for example (Rutherford, 2014).

“While over 60 percent of the carcass can find its way into lower value or ground products, the production model requires most of these animals to be fed an expensive ration that aims to perfect the quality of, at best, 30 percent of the carcass. Essentially, the industry is producing an extraordinarily high-grade product for consumers who wish to purchase a commodity,” (Close, 2014; Rutherford, 2014).

## Where to Go From Here?

While plant-based meat analogs and lab-cultivated muscle tissue certainly qualify as disruptive technologies in the meat industry, the markets are as-yet tiny compared with the size of the traditionally raised and harvested meat. As production on the new products picks up steam, the costs of production are dropping to a point where regular consumption, at least in developed countries, makes sense. (As of early 2018, for example, the upscale Umami Burger chain was menuing the Impossible Burger for a wallet-gouging $16 per sandwich. At White Castle, on the other hand, the Impossible Burger sliders are priced at $1.99 each.)

The meat industry has the chance to defend its territory, however. A first step has been taken by the meat organizations seeking clarification on how regulators plan to treat these products. Companies have additional options, such as further processing (slicing, dicing, cubing, grinding) meats so they can be more easily combined as an ingredient in dishes by consumers who have less and less time to cook. Marinating and otherwise pre-prepping meats to be cooked also is appealing to time-starved shoppers.

While the fact that 60% of meat consumed in the United States is eaten in the form of ground beef points to the profitability of focusing on commodity meats, niches for certified Organic, “all-natural” and grass-fed meats, heritage pork, and so-called slow growth chicken breeds, while small, are seeing rocketing growth. They are a way for companies to address the meat analogs and cultured tissue’s claims of greater environmental awareness and even health by giving the meat a health or environmental “halo” of its own.

The meat industry will lose some volume to these new technologies, that’s certain, but the next 3 to 5 yr will tell the tale: Will the ground lost be negligible or damaging? Will the industry find ways to fill new niches with traditionally harvested meat even as consumers go for the novelty appeal of the new products? Will companies be able to export their products at least as often and in volumes as they do now, or will meat products get caught in a web of tariffs and retaliation globally?

**Cellular Agriculture – Growing “meat” in the lab**

To produce “cultured meat” in the lab requires muscle tissue from an animal. A small amount of muscle is surgically removed by a needle biopsy, and the muscle cells are enzymatically separated from the tissue sample to obtain myoblasts. These cells are muscle stem cells (satellite cells) that are able to proliferate in culture through a few dozen rounds of cell division to produce new muscle cells.

**Figure:**
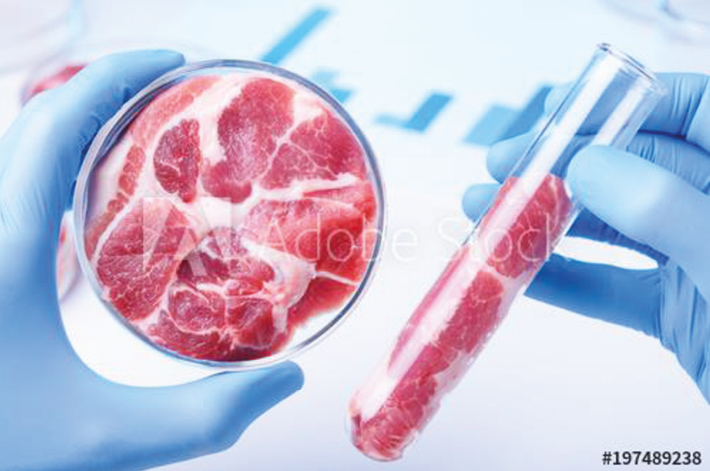
Meat in laboratory test tube and in lab Petri dish, cultured clean laboratory meat concept. (source: Adobe stock image)

Myoblasts require a surface for attachment, such as a specially coated plastic flask or a scaffolding of collagen or other material, in order to grow and form small muscle fibers (less than 1 mm diameter). The cells in the culture are grown in a liquid growth medium that includes required nutrients sourced from serum from fetal calves. For large scale production, current challenges remain for automating the process, and scaling production to volume so that cells can be mass produced in large bio-reactor vats.

For Mark Post’s lab-burger taste test, after 3 months of culture, 20,000 strands of fiber were assembled into a burger, along with beet juice for color. The companies with various lab meat projects in development are working to replace the serum with individual nutrients, but this process, so far is much less efficient and more costly than serum. They are also growing fat cells (adipocytes) to mix with the muscle fibers to improve flavor, and will use the myoglobin that give meat its red color instead of beet juice. In order to move beyond products that simulate ground meat, developers will also have to incorporate connective tissue and other structural features.

(Arshad et al., 2017; Post and Hocquette, 2017)

**Figure:**
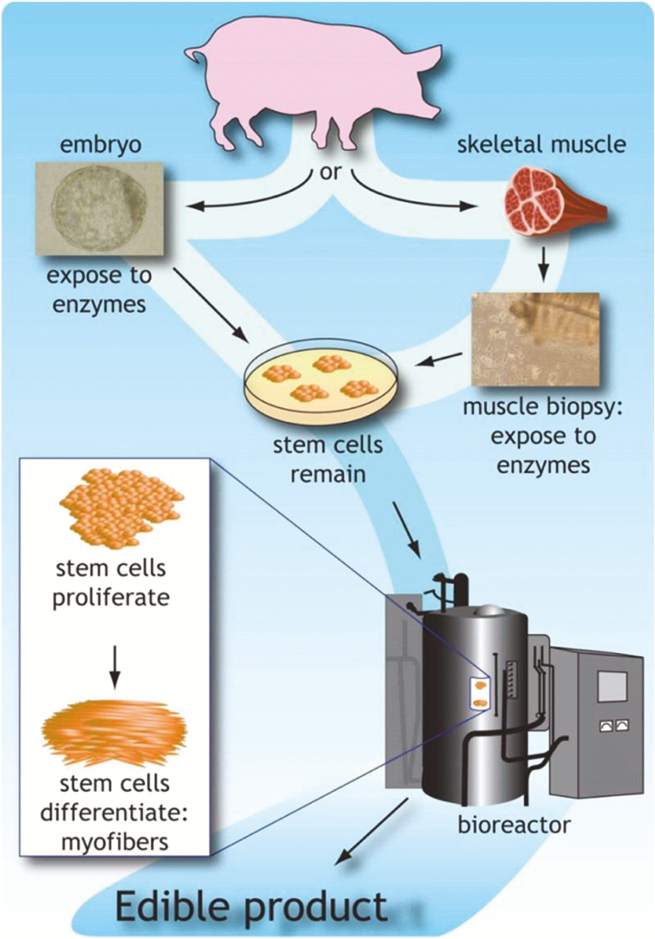
Meat in laboratory test tube and in lab Petri dish, cultured clean laboratory meat concept. (source: Adobe stock image)

In Vitro Meat: This is Figure 4 taken from“Promise and Ontological Ambiguity in the In vitro Meat Imagescape: From Laboratory Myotubes to the Cultured Burger”

Neil Stephens & Martin Ruivenkamp

https://doi.org/10.1080/09505431.2016.1171836

https://www.tandfonline.com/doi/full/10.1080/09505431.2016.1171836

## About the Author

**Ms. Keefe** joined the Meatingplace team as editor 10 years ago and has overseen an increase in readership and interest in the publication both within and outside the meat industry. Under her guidance, the publication has brought home 20 national awards, including Jesse H. Neal National Business Journalism Awards and Azbees, awarded by the American Society of Business Press Editors, recognizing its exemplary coverage of the industry. In addition to overseeing all content in the monthly magazine, Keefe writes the Center of my Plate blog on Meatingplace.com, covers breaking and daily news items for the Meatingplace newsletter, produces and edits Meatingplace videos, and speaks publicly on all things meat. Prior to joining Meatingplace, Keefe was editor of the American Marketing Association’s flagship magazine, Marketing News, and worked at Crain’s Chicago Business, The Orlando (FL) Sentinel and Forbes magazine. Keefe earned bachelor’s and master’s degrees from Northwestern University’s Medill School of Journalism.
